# Renal Dysfunction in Post-Stroke Patients

**DOI:** 10.1371/journal.pone.0159775

**Published:** 2016-08-30

**Authors:** Kamil Chwojnicki, Ewa Król, Łukasz Wierucki, Grzegorz Kozera, Piotr Sobolewski, Walenty M. Nyka, Tomasz Zdrojewski

**Affiliations:** 1 Department of Neurology, Medical University of Gdańsk, Gdańsk, Poland; 2 Department of Nephrology, Medical University of Gdańsk, Gdańsk, Poland; 3 Department of Arterial Hypertension and Diabetology, Medical University of Gdańsk, Gdańsk, Poland; 4 Department of Neurology and Stroke Unit, Hospital of Sandomierz, Sandomierz, Poland; Hospital Universitario de la Princesa, SPAIN

## Abstract

**Background:**

The presence of chronic kidney disease (CKD) is an indicator of a worse long-term prognosis in patients with ischemic stroke (IS). Unfortunately, not much is known about renal function in the population of post-IS subjects. The aim of our study was to assess the prevalence of renal damage and impaired renal function (IRF) in the population of post-IS subjects.

**Methods:**

This prospective analysis concerned 352 consecutive post-IS survivors hospitalized in Pomeranian stroke centers (Poland) in 2009. In this group estimated glomerular filtration rate (eGFR) according to MDRD (modification of diet in renal diseases) and CKD-EPI (Chronic Kidney Disease Epidemiology Collaboration) formulas and urine albumin/creatinine ratio (ACR) were determined.

**Results:**

Among survivors decreased eGFR (<60 mL/min./1.73m^2^ according to MDRD or CKD-EPI) or ACR≥30mg/g were detected in 40.38% (23.07% Men, 55.32% Women; *P<0*.*01*). The highest prevalence of IRF was noted in post-IS subjects with atheromatic and lacunar IS. In multivariate analysis the ACR≥30mg/g was predicted by older age, diabetes mellitus (DM) and physical disability (modified Rankin scale 3–5 pts.). The association with reduced eGFR was proved for sex (female), DM and physical disability.

**Conclusions:**

CKD is a frequently occurring problem in the group of post-IS subjects, especially after lacunar and atheromatic IS. Post-IS patients, mainly the elderly women, with physical disability and diabetes mellitus, should be regularly screened for CKD. This could reduce the risk of further cardiovascular events and delay the progression of IRF.

## Introduction

Worldwide, stroke is the second most common cause of mortality and the third most common cause of disability [1].

Moreover, the incidence of stroke is increasing in Eastern Europe while, in contrast, in the US and Western Europe the incidence of stroke is decreasing [2].

The prevalence of Chronic Kidney Disease (CKD) worldwide is continuously increasing (data from Fresenius Medical Care and from United States Renal Data System). However, a credible assessment of the occurrence of CKD is limited, apart from stage 5, where data is quite precise due to the need for renal replacement therapy [3–5].

Patients with CKD have an increased risk of cardiovascular diseases (CVD). The increased CVD risk in patients with end-stage renal disease (ESRD) with eGFR below 15 mL/min/1.73 m^2^ has been well described, but there is now clear evidence that mild to moderate renal dysfunction is also associated with a substantial increase in CVD risk [6].

Practice guidelines from the National Kidney Foundation in 2002 recommended that CKD should be considered as CVD risk equivalent [7].

Both decreased glomerular filtration rate and the presence of albuminuria/proteinuria, increase the risk of CVD. These associations have been shown in community-based populations (ie, cohorts that were not selected specifically to enroll individuals with CKD or CVD), and in populations of patients at high cardiovascular risk (ie, cohorts in which patients with pre-existing CVD or CVD risk factors were specifically recruited) [8,9]

Impaired renal function is an independent risk factor of IS. Furthermore, the presence of CKD is an indicator of a worse long-term prognosis in post-IS patients.

Unfortunately, not much is known about renal function in the population of post-IS subjects. The data concerning the prevalence of CKD in this group is very limited. Earlier studies were conducted among different populations (eg. Caucasian or not Caucasian) and stroke subtypes (ischemic, hemorrhagic or transient ischemic attack). Furthermore, in those surveys the way of renal impairment estimation was not the same [10–15]. The estimation of renal impairment was varied.

The aim of the study was to assess the prevalence of renal damage and impaired renal function in the population of post-IS subjects beyond the acute phase of stroke. An additional aim was to estimate the need for professional nephrologic care in this group of patients.

## Patients and Methods

All post-IS subjects were recruited to the study after written or verbal (in case of dominant hand dysfunction) informed consent.

The study was ethically approved by bioethical committee of Medical University of Gdańsk (Poland).

### Source of data

Diagnosis of IS was based on the WHO’s definition and stroke etiology according to the TOAST classification [16, 17].

Patients' medical and demographic records were obtained from the Pomeranian Stroke Register (PRUM). This is an open, multi-center, Internet-based, consecutive stroke register established in 2006 at the Neurology Department of the Medical University of Gdańsk, in Gdańsk, Poland). Detailed characteristics of PRUM were already presented in an earlier publication [18].

### Recruitment and study organization

The study concerned post-IS patients > = 18 years old who had been hospitalized during the period of January-March 2009 in randomly selected 3 out of 15 stroke centers in Pomerania. The study was conducted beyond acute phase of stroke to ensure the reliability of measurements (renal formulas requires that renal function is in a steady state).

The survey fieldwork was carried out in the period of 3 to 6 months after IS by nurses working at neurological wards, who were familiar with IS-subjects and who were experienced in performing such tests in the elderly (over 60 years old). The nurses had been trained by the authors of the project to assess the functional and mental status of the patients. The training also concerned interpersonal techniques, aimed at reducing the percentage of potential refusals to participate in the study.

A nurse visited the patient in their house two to three times.

During the first visit she collected the medical history of the patient and completed a questionnaire. She also performed a functional assessment, anthropometric and blood pressure measurements.

The second visit was to repeat blood pressure measurements and to collect blood and urine samples. Patients with increased fasting glucose (without previously diagnosed diabetes mellitus) had an additional glucose level measurement during the third visit.

To compare surviving and non-surviving subjects the data from hospital records was used. Laboratory results were derived from last in-hospital sampling.

### Questionnaire

A detailed questionnaire was used to evaluate several aspects, namely: comorbidity, treatment methods, lifestyle and knowledge of cardiovascular prevention among the subjects as well as their socio-demographic status.

The questionnaire was completed on the basis of medical history collected from either the patient or their guardian.

### Functional assessment

Functional status (FS) was rated during the first visit. Modified Rankin score (mRs) and Barthel Index (BI) were used to assess FS [19]. Potential cognitive impairment was screened by Mini-Mental State Examination (MMSE) [20]. Initial severity of stroke was rated in PRUM with National Institutes of Health Stroke Scale (NIHSS).

### Measurements

Anthropometric measurements were performed during the study: height, body weight, waist, hip and arm size, blood pressure (BP) and pulse. The blood pressure was measured twice on the operational or on the right arm (if both arms were operational) with the automatic Omron M5I apparatus (Omron, Hoffman Estates, USA).

Arterial hypertension (AH) diagnostics was performed on the basis of two measurements during two independent visits, in accordance with JNC VII guidelines [21].

### Blood and urine sampling and renal function estimation

Blood and urine sampling was completed in the respondents’ homes during the second visit. All analyses were performed in the Central Laboratory of Medical University of Gdansk. The Laboratory of Gdansk’ Medical University is encompassed in the international Common External Quality Assessment System LabQuality. The measurements were taken with the help of the Architect c8000 chemistry analyser (Abbott Laboratories, Abbott Park, USA). Creatinine was measured using the Jaffe method. The concentration of albumin in the urine was expressed as for every 1g of excreted creatinine. The albumin/creatinine urinary ratio was calculated. Renal function was estimated by eGFR with MDRD and CKD-EPI formulas. The concentrations of total cholesterol, triglycerides and HDL cholesterol were measured using enzymatic methods. The fasting glucose in venous blood was measured with the enzymatic/hexokinase and glucose-6-phosphate dehydrogenase reaction.

### Statistical analysis

Statistical analyses were performed using the SAS System v. 9.4 software (SAS, Carry, USA). Continuous variables are expressed as mean and standard deviation (SD) or median. Categorical variables are expressed as a percentage. Simple associations were performed using t-test or appropriate non-parametric tests for continuous variables, and chi-square tests for categorical variables. The stepwise logistic regression models were used to identify the variables from univariate analyses predicting renal dysfunction or death among post-IS subjects. In this case, odds ratios (OR), and 95% confidence intervals (CIs) were generated. The variable entry criterion to models was set to a significance level of *P* ≤ 0.25 for any ratio calculation from univariate analysis, and the variable retention criterion to *P* < 0.05.

## Results

Originally, the survey was to include 597 patients who survived hospitalization due to IS. Overall, 352 subjects participated in the project (66.42% of survivors), including 191 men (M) and 161 women (W). Blood and urine tests were performed in 306 people. Table 1 shows the details of the recruitment process.

**Table 1:** Description of recruitment to the study.

Status description	N	%
All patients hospitalized due to IS in selected stroke units	660	100,0
Respondents deceased during hospitalization	63	9.5
All subjects surviving hospitalization in selected centers	597	90,5
Respondents deceased between the end of hospitalization and first visit	67	10,2
All patients provided informed consent	352	53,3
Visits I, II or I, II, III (blood and urine testing performed)	306	46,4
Visits I and II, no blood and urine testing (samples not properly analyzed by central laboratory due to pre-laboratory or laboratory errors)	29	4,4
Visit I only, no blood and urine sampling (no informed consent for sampling)	17	2,6
Refusals	100	15,2
Wrong addresses	55	8,3
No information	23	3,5

Median time from IS to the first assessment was 4.0 months (mean– 4.31, SD 1.85). The baseline characteristics of the examined population (the surviving and non-surviving, respectively), based on data from hospitalization period, are shown in Table 2.

**Table 2:** Baseline characteristics and comorbidities of surviving and non-surviving subjects with ischemic stroke (data derived from hospitalization period).

			Surviving subjects (N = 597)			Non-surviving subjects (N = 130)		
			All	Women	Men	All	Women	Men
	
**Age (mean± SD)** ^1^ ^2^ ^3^		[years]	69.94 ±12.42	73.96 ±11.93	66.49 ±11.78	74,62 ±11.74	76,54 ±9.59	71,69 ±14.09
**NIHSS at admission to stroke unit (mean± SD)** ^1^		[pct]	11.27 ±4.98	11.04 ±4.84	11.43 ±5.08	16.30 ±4.85	16.84 ±5.19	15.48 ±4.23
**Serum creatinine**^1^		μmol/L	84.86 ±25.64	90.17 ±23.00	79.56 ±28.29	114.04 ±40.66	123.76 ±35.17	99.01 ±43.36
**eGFR (mean± SD)**	**MDRD**^1^,^2^,^3^	mL/min/ 1.73m^2^	78.21 ±21.58	70.29 ±21.40	84.71 ±21.70	70.02 ±26.54	62.35 ±27.24	82.78 ±22.58
	**CKDEPI**^1^,^2^,^3^	mL/min/ 1.73m^2^	73.94 ±19.47	67.10 ±20.52	79.55 ±18.44	68.28 ±21.37	60.43 ±22.80	74.33 ±16.67
**Arterial hypertension** ^3^		[%]	81.48	83.75	79.58	81.23	87.27	72.07
**Diabetes mellitus**		[%]	31.05	34.38	28.27	31.92	35.00	27.24
**Hypercholesterolemia**		[%]	60.11	61.88	58.64	69.86	68.18	72.41
**BMI≥25 kg/m**^**2**^		[%]	58.24	62.30	53.75	no data	-----	-----
**Atrial fibrillation** ^1^		[%]	27.64	32.50	23.56	53.42	54.55	51.72
**Smoking (active/ex)** ^2^ ^3^		[%]	59.26	35.00	79.58	55.07	46.82	67.59

eGFR—estimated glomerular filtration rate; MDRD—modification of diet in renal diseases formula; CKD-EPI—Chronic Kidney Disease Epidemiology Collaboration formula; BMI—body mass index; MRs—modified Rankin scale;

^1^—P<0.05 survivors vs deaths (all)

^2^—P<0.05 women vs men (surviving)

^3^—P<0.05 women vs men (non-surviving)

The average age of survivors was nearly 5 years lower than that of the deceased patients (p<0.05). In both groups women were definitely older than men. There was a high prevalence of CVD risk factors especially AH and HCH.

Initial severity of stroke measured with NIHSS at admission was higher among the non-surviving group. A significant degree of physical disability (mRS 3 to 5 pts.—incapable of independent existence) was noted in over 50% of the surviving and 90% of the non-surviving subjects (p<0.05). In the non-surviving group there was much higher proportion of cardiogenic stroke (in more than a half of subjects IS was due to atrial fibrillation).

Impaired renal function (eGFR<60mL/min/1.73m^2^) was found in 18.0% of the surviving patients when MDRD formula was used, and in 20,9% according to the CKD-EPI formula. Corresponding numbers for non-surviving subjects were 35,62% (MDRD) and 43,84% (CKD-EPI).

### Analysis of the surviving patients data (nurses visits)

According to TOAST classification, 36.52% of the examined IS survivors had a large-artery atherosclerosis (embolus/thrombosis), 25.51% were diagnosed with a small-vessel occlusion (lacunar infarcts), and 23.48% of the patients suffered from cardiac thromboembolism. In 14.49% of the remaining subjects the etiology was other or undetermined. More than 1/3 of survivors showed a substantial cognitive impairment (MMSE<24).

**Fig 1:**
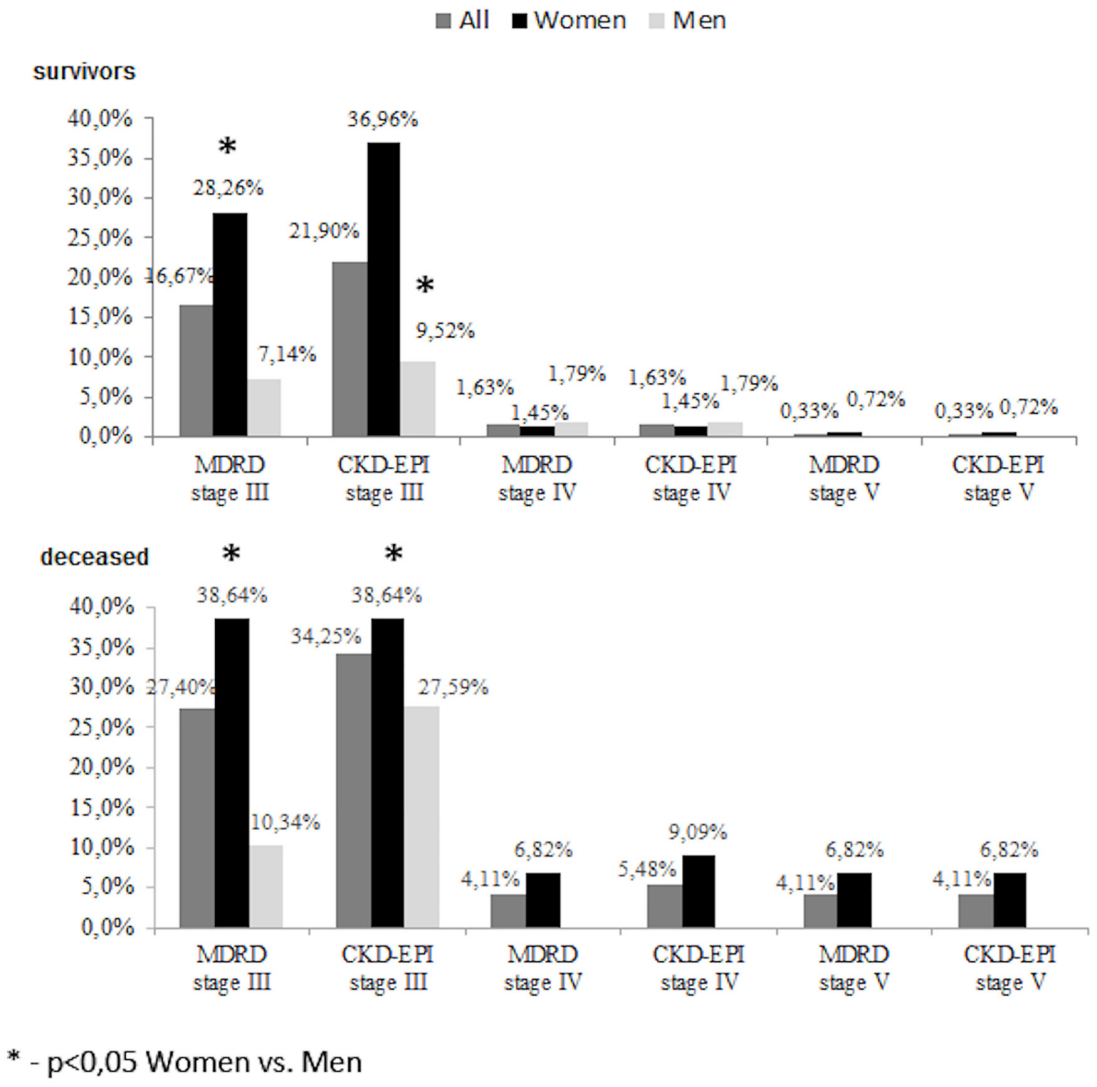
Distribution of stage 3 to 5 of chronic kidney disease on the basis of glomerular filtration rate measured according to MDRD and CKD-EPI formulas in post-IS subjects.

Impaired renal function (eGFR<60mL/min/1.73m^2^) was diagnosed in 18.63% of the surviving patients (W 30.43% vs. M 8.93%, *P<0*.*01*) when MDRD formula was used, and in 23.86% according to the CKD-EPI formula (W 39.13% vs. M 11.31%, *P<0*.*01*). These results didn’t significantly differ from those obtained (from)/during hospitalization period. The incidence of the eGFR of stage 3 to 5 is shown in Fig 1.

Urine albumin/creatinine ratio (ACR)≥30 mg/g was noted in 27.62%. It was significantly more frequent in women than in men (34.15% vs. 22.70% respectively, *P = 0*.*03*). Albuminuria≥30 mg/l was detected in 27.12% of post-IS subjects.

Impaired renal function (eGFR<60 mL/min./1.73m^2^ according to MDRD or CKD-EPI or ACR≥30 mg/g) was present in 40.38% of the surviving subjects (23.07% M, 55.32% W; *P<0*.*01*).

**Fig 2:**
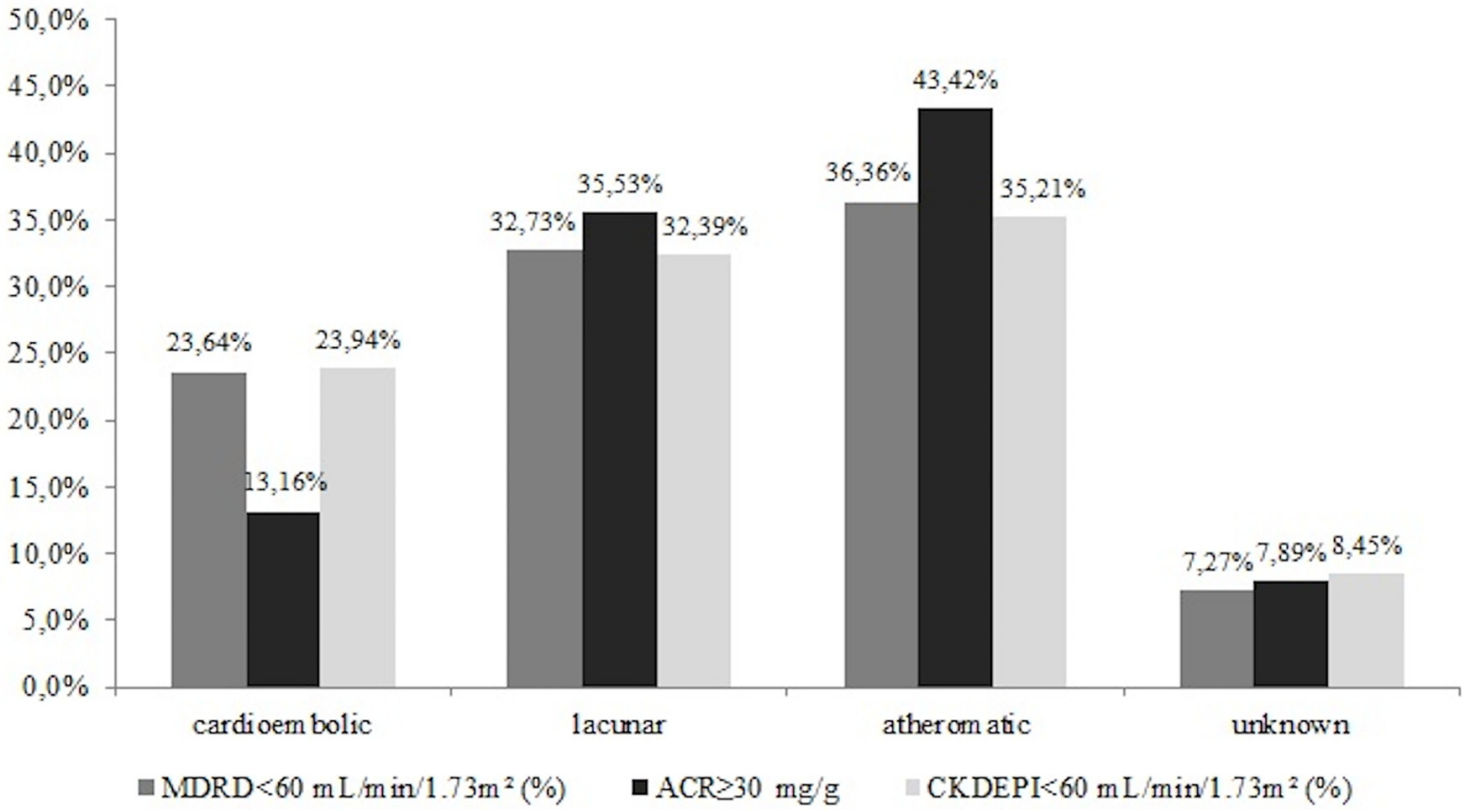
Frequency of diminished kidney function (eGFR < 60 mL/min/1.73m^2^) estimated by MDRD and CKD-EPI formulas and albuminuria measured as urine albumin to creatinine ratio (ACR) in survivors with ischemic stroke according to IS etiology.

The incidence of ACR≥30 mg/g in patients with lacunar or atheromatic stroke was significantly higher than in the group of cardioembolic IS (Fig 2). A similar relationship was noted for reduced eGFR (<60 mL/min/1.73 m^2^ for both—MDRD and CKD-EPI formulas).

Univariate analysis of potential risk factors for albuminuria (ACR)/impaired filtration is shown in Table 3.

**Table 3:** Univariate analysis for potential risk factors of chronic kidney disease in examined population of surviving subjects with ischemic stroke (data from nurses visits).

		ACR≥30 mg/g (%)			MDRD <60mL/min/1.73m^2^ (%)			CKD-EPI <60mL/min/1.73m^2^ (%)		
		All	M	W	All	M	W	All	M	W
**Age group**	**<65**	12.62	13.33	10.71	2.78	0.00	10.00	1.85	0.00	6.67
	≥65	35.87	30.68	41.05	27.27	16.67	36.11	35.86	21.11	48.15
	*P*	<0.01	<0.01	<0.01	<0.01	<0.01	<0.01	<0.01	<0.01	<0.01
**Education**	Primary /vocational	33.16	27.84	38.39	20.9	10.31	30.77	26.37	12.37	39.42
	Secondary /higher	17.17	15.15	21.21	14.71	7.14	31.25	18.63	10.00	37.50
	*P*	<0.01	0.06	0.07	0.19	0.48	0.96	0.14	0.63	0.84
**MRs**	3–5	36.36	27.27	47.27	25.37	15.15	35.29	32.84	18.18	47.06
	0–2	20.00	18.28	22.81	11.69	4.12	24.56	14.94	6.19	29.82
	*P*	<0.01	0.18	<0.01	<0.01	0.01	0.19	<0.01	0.02	0.04
**Cognitive impairment**	MMSE≥24	21.34	21.00	21.88	14.37	5.88	27.69	19.16	7.84	36.92
	MMSE<24	31.68	24.53	40.43	25.45	14.55	36.36	31.82	18.18	45.45
	*P*	0.06	0.62	0.04	0.02	0.07	0.31	0.02	0.05	0.34
**Arterial hypertension**	No	22.45	18.75	31.25	16.67	9.38	27.27	22.22	9.38	40.91
	Yes	28.57	23.66	34.58	19.05	8.82	31.03	24.21	11.76	38.79
	*P*	0.38	0.55	0.79	0.68	0.92	0.72	0.76	0.70	0.85
**Diabetes mellitus**	No	21.94	22.03	21.79	16.1	9.24	25.58	20.98	11.76	33.72
	Yes	39.56	24.44	55.56	23.76	8.16	38.46	29.7	10.02	48.08
	*P*	<0.01	0.74	<0.01	0.07	0.82	0.11	0.09	0.77	0.09
**HCH**	No	32.08	25.00	42.86	19.83	10.61	32.00	27.59	12.12	48.00
	Yes	24.86	21.21	29.63	17.89	7.84	29.55	21.58	10.78	34.09
	*P*	0.19	0.57	0.14	0.67	0.54	0.76	0.23	0.79	0.11
**Atrial fibrillation**	No	29.89	23.26	36.36	22.58	8.94	35.42	30.11	12.20	50.00
	Yes	26.50	22.50	32.91	16.9	8.89	27.78	21.13	8.89	33.33
	*P*	0.56	0.92	0.70	0.24	0.99	0.35	0.12	0.55	0.06
**BMI**	<30 kg/m^2^	27.14	23.36	31.52	16.67	8.18	26.00	22.38	10.91	35.00
	≥30 kg/m^2^	26.87	21.74	38.10	20.59	6.25	55.00	22.06	8.33	55.00
	*P*	0.97	0.83	0.56	0.46	0.67	0.01	0.96	0.62	0.09
**Smoking status**	smokers (active/ex)	22.94	22.66	24.39	12.43	9.02	22.73	14.69	11.28	25.00
	non-smokers	34.19	22.86	39.02	27.13	8.57	34.04	36.43	11.43	45.74
	*P*	0.04	0.98	0.11	<0.01	0.93	0.18	<0.01	0.98	0.02
**ACEI/ARBs therapy**	No	32.39	24.05	43.55	20.00	10.98	30.88	26.00	14.63	38.57
	Yes	22.76	21.43	24.59	17.31	6.98	30.00	21.79	8.14	39.71
	*P*	0.07	0.67	0.03	0.32	0.36	0.91	0.23	0.18	0.89

Among women, statistical links to ACR≥30 mg/g were found for: age ≥65, functional dependency (mRs 3–5 pts.), presence of cognitive impairment (MMSE<24 points), diabetes mellitus, the lack of ACEI/ARBs drugs in the therapeutic regimen. For men major factor was only age ≥65 years.

Impaired glomerular filtration in women was related to age ≥ 65 years, mRs 3–5 points, no smoking and obesity (BMI> = 30 kg/m2). Among men impaired renal function was not influenced by modifiable CVD risk factors (including AH). However, it was related to age ≥65 years and mRs 3–5 points.

To better characterize independent risk factors for renal impairment a multivariate logistic regression analysis was performed. The multivariate analysis predicted respectively:

1. the occurrence of reduced eGFR (MDRD<60 or CKD-EPI<60 mL/min/1.73m^2^);
2. the occurrence of albuminuria (ACR≥30 mg/g).

Independent variables for these stepwise logistic regression models were selected from Table 3 (columns “All”). The predictors of reduced eGFR were: sex (female), older age and diabetes mellitus. For elevated ACR significance level *<0*.*05* was proved for older age, diabetes mellitus and mRs 3–5 points (Table 4).

**Table 4:** Stepwise logistic regression models for potential risk factors of chronic kidney disease in examined population of surviving subjects with ischemic stroke (based on results from nurses visits).

Effect	ACR≥30 [mg/g]			MDRD<60 or CKD-EPI<60 [mL/min/1.73m^2^]		
	OR	95% CI	*P*	OR	95% CI	*P*
**Sex (female)**	1.01	0.49–2.06	0.98	3.43	1.40–8.36	0.01
**Age (every 5 years)**	1.37	1.10–1.72	<0.01	2.01	1.39–2.90	<0.01
**Higher education**	0.60	0.30–1.18	0.14	0.87	0.37–2.04	0.74
**MRs 3–5**	2.40	1.71–2.76	0.03	1.56	0.69–3.50	0.28
**MMSE<24**	0.83	0.41–1.68	0.60	0.83	0.36–1.93	0.66
**Diabetes mellitus**	2.19	1.16–4.15	0.02	2.59	1.74–3.43	0.03
**Hypercholesterolemia**	0.92	0.48–1.76	0.80	0.87	0.40–1.90	0.73
**Atrial fibrillation**	---------	------------	-------	1.20	0.54–2.64	0.66
**ACEI or ARBs therapy**	0.69	0.37–1.27	0.23	0.68	0.32–1.40	0.29
**Smoking**	1.03	0.48–2.20	0.94	1.90	0.76–4.76	0.17

a P value of <0.05 is considered statistically significant; mRs—modified Rankin scale; MMSE—mini-mental state examination; ACEI—angiotensin-converting-enzyme inhibitor; ARBs—Angiotensin II Receptor Blockers.

In order to analyze the impact of renal dysfunction on mortality during the period from IS to the first visit, a multivariate model was built based on Table 2. Besides eGFR<60 mL/min/1.73m^2^, the model included sex, age intervals (every 5 years), atrial fibrillation and ranges of NIHSS value (every 4 points). In this model of a stepwise logistic regression the reduced eGFR wasn’t a statistically significant risk factor of post-IS mortality (OR 1.83, 95% CI 0.96–3.50; *P = 0*.*06*), but the model was strongly influenced by higher NIHSS (OR 2.05; 95% CI 1.64–2.57; *P<0*.*01*) and older age (OR 1.29; 95% CI 1.04–1.59; *P = 0*.*02*) in non-survivors.

## Discussion

The results of our study support the hypothesis that CKD is a frequently occurring problem in the group of post-IS subjects.

Both CKD and IS are issues of increasing importance not only in Poland, but also in Eastern Europe. This is due to a high incidence rate and a slow decrease in mortality rate, comparing to the countries of the so-called old EU.

Our study shows a significantly higher prevalence of renal dysfunction in the post-IS population as compared to the general population. The biggest difference concerns a higher incidence of albuminuria: 27.1% in our study vs. 11.9% in the general population (PolNef study, n = 2471, age range 18–98) [22].

According to our study the frequency of renal dysfunction among post-IS patients definitely increases with age. Similar results come from PolSenior survey (Poland, n = 3793, age range 65–104) and from NHANES 1999–2006 (USA, n = 16032, age range ≥20) [23, 24].

In the PolSenior study the incidence of ACR≥30 mg/g was lower than in the post-IS population (12.5% vs 27.6%) However, the average age in the PolSenior study was about six years higher (76 vs 70 years) [23]. Compared with the data from NHANES, the incidence of albuminuria in our study was almost 3 times higher [24].

In our study, urine albumin concentration was compared/likened to urine creatinine concentration, which made the albuminuria result unaffected by the amount of excreted urine (in this way, the results are not dependent upon urine specific gravity). Ultimately, however, in our study, ACR ≥ 30 mg/g and albuminuria ≥ 30 mg/L occurred with the same frequency.

Similarly to albuminuria, also reduced filtration occurred more frequently in post-IS patients. The filtration <60 mL/min/1.73m^2^ (MDRD formula) occurred in 18.6% of post-IS patients vs. 8.8% in the general population (PolNef Study) [25]. A proportion of the reduced eGFR in our study was also higher than among white participants of REGARS Study (n = 16410, age ≥ 45, eGFR<60 mL/min/1.73m^2^—from 4.3 to 16.7%) [25]. It should be mentioned that post-IS subjects, especially in mRs 4 and 5 points are often malnourished and equations using creatinine for GFR calculation can be far from measured GFR.

On the basis of the Report of Renal Replacement Therapy in Poland it is known that renal replacement therapy in Pomeranian Region in 2008 was implemented among 40/100 thousand patients. In our study the occurrence of end-stage renal disease (eGFR<15 mL/min/1.73m^2^) was 0.33%, and does not differ much from the general population of Poland [26]. However, it should be remembered that even in an early stage of CKD the risk of cardiovascular complications increases considerably, hence the need to actively screen the post-IS population for CKD [27]. The rate of ESRD in our study would likely be higher provided longer follow-up. According to Taiwan National Health Insurance program, during 4-year follow-up this rate achieved 1,77% among post-IS subjects without previous CKD [28].

An early professional therapy of CKD in post-IS subjects could reduce the risk of recurrent strokes which are usually more severe and generate greater medical and social cost (the risk of recurrent stroke reaches 40% within 5 years) [6, 29].

Our results show a statistical link between certain factors and renal impairment in post-IS population. In multivariate analysis albuminuria was predicted by older age, DM and worse functional status (mRs 3–5). The reduced eGFR was influenced by older age, sex (female) and DM. These results are partially consistent with other surveys like PolSenior and Framingham Heart Study [23, 30]. The disability seems to be rather a consequence of renal damage—there is strong evidence that renal failure has a negative impact on post-IS prognosis (patients with kidney dysfunction have higher mortality and higher mRs scoring, especially due to motor deficit [10, 11, 31, 32]. In our study renal impairment has no effect on overall mortality in multivariate analysis, but the model is strongly influenced by age and severity of stroke. It should be noted that the comparison of the surviving and non-surviving patients must be interpreted with caution because the evaluation of the deceased group was incomplete and retrospective (initially they did not account for testing). Clinical and biochemical parameters of the group were derived from the acute phase of stroke, which limits their value.

Our study shows the prevalence of renal dysfunction among post-IS survivors in Pomeranian Region (Poland). This is the only such analysis on the prevalence of CKD in the post-IS population. Other existing analyses were generally observational (eg. HOPE—Canada) or included hemorrhagic stroke. They were also commonly based on renal assessment in acute phase of stroke, when the value of GFR formulas may be limited. In comparison with our results, studies of Tsagalis et al. (Grece) or Yahalom et al. (Israel) conducted in acute phase of stroke showed slightly greater proportion of reduced eGFR (respectively 28% and 22% of patients with eGFR<60 mL/min/1.73m^2^ according to MDRD formula) but they concerned both ischemic and hemorrhagic stroke [10–13]. In the study of Tsakamoto et al. the prevalence of reduced eGFR was 38% (non Caucasian acute stroke population including also subjects after transient ischemic accidents—TIA) [14].

A retrospective study of Y. Bao (China) on IS patients (data from acute phase) analyzed a slightly larger group than ours. However, albuminuria or ACR were not specified there, CKD-EPI formula was not used, and the prevalence of end-stage renal disease was not estimated (eGRF<15 mL/min/1.73m^2^). There was also no analysis based on the etiology of IS. Significantly fewer patients from the Chinese study were in the eGFR range of 15–59 mL/min/1.73m^2^ (<10% vs. 17.6% in our study). This difference may be also due to a lower average age of patients (by nearly 8 years) in the Y. Bao et al. study [15].

The results obtained from our study indicate a higher incidence of renal dysfunction in the case of a lacunar or atheromatic IS. This relationship seems rather logical, since it is known that both small vessel disease and atheromatic macroangiopathy result in the damage of the kidney and brain. Especially cerebral small vessel disease (SVD) is closely associated with kidney function in patients with stroke [33,34].

Our data do not show clear evidence of a higher incidence of arterial hypertension in CKD group compared with the group without CKD. The incompatibility of our results with the previous publications may result from:

1. high prevalence of AH (81%) in the examined population—the group without AH was small, which probably made it difficult to detect statistical significance.
2. a considerable number of patients taking ACEI/ARBs drugs; 58% of the examined patients took these drugs, which decreased albuminuria.

In conclusion, CKD is a common disorder in the post-IS patients, especially after lacunar and atheromatic IS. Subjects after IS, mainly the elderly (>65 years old), in a worse functional status (mRs>2), with diabetes mellitus, should be regularly screened for CKD. In the presence of abnormalities they should be professionally treated. This would reduce the risk of subsequent cardiovascular events and delay the progression of renal dysfunction.
